# Supplementary material for: Mark–Release–Recapture Trial with Aedes albopictus (Diptera, Culicidae) Irradiated Males: Population Parameters and Climatic Factors
Source: Insects. 2024 Sep 11;15(9):685. doi: 10.3390/insects15090685 (PMC11432691; doi:10.3390/insects15090685)
Supplement: Supplementary file 1 [file insects-15-00685-s001.zip › insects-3148099-supplementary.pdf]

Supplementary Table S1: Association between marking colours and distance and capture

	OR	95% CI	p-value
**Blue**			
**Distance**	1.04	0.72, 1.50	0.83
**Capture**	1.06	0.97, 1.15	0.20
**Orange**			
**Distance**	1.03	0.70, 1.52	0.89
**Capture**	1.04	0.95, 1.14	0.35
**Pink**			
**Distance**	1.08	0.75, 1.56	0.67
**Capture**	**1.09**	**1.01, 1.18**	**0.03**
**Red**			
**Distance**	1.08	0.75, 1.55	0.69
**Capture**	**1.09**	**1.01, 1.18**	**0.04**
**Yellow**			
**Distance**	1.07	0.74, 1.55	0.71
**Capture**	**1.10**	**1.02, 1.19**	**0.02**

OR: odds-ratio, CI: confidence interval. Significant values are displayed in bold.

Supplementary Table S2: Summary of the climatic conditions during human landing collection.

Variable	Statistics[1]
Temperature	21.95 (1.04)
Wind intensity (in km)	12.4 (4.9)
Wind intensity categorised (in km)	
Weak	560 (63.5%)
Moderate	322 (36.5%)
Wind direction	
---	0 (0.0%)
N	12 (1.4%)
NE	0 (0.0%)
E	120 (13.6%)
SE	238 (27%)
S	12 (1.4%)
SW	238 (27%)
W	230 (26.1%)
NW	32 (3.6%)
Cumulative precipitation categorised	
None	34 (3.9%)
Precipitation	848 (96.1%)
Humidity (%)	81 (10)

[1] For continuous variables the mean and standard deviation is presented and for categorical variables, the absolute and relative frequency.

Supplementary Table S3: Association between release rounds and climatic conditions.

	OR	95% CI	p-value
**First round**			
**Wind intensity (ref. weak)**	**0.00**	**0.00, 0.01**	**<0.001**
**Temperature (centered at the mean)**	1.70	0.93, 3.10	0.086
**Humidity (centered at the mean)**	**0.81**	**0.77, 0.86**	**<0.001**
**Precipitation (ref. none)**	**0.00**	**0.00, 15,111,808,362**	**0.7**
**Third round**			
**Wind intensity (ref. weak)**	**0.01**	**0.00, 0.01**	**<0.001**
**Temperature (centered at the mean)**	**5.20**	**2.81, 9.63**	**<0.001**
**Humidity (centered at the mean)**	0.96	0.91, 1.02	0.2
**Precipitation (ref. none)**	**22.7**	**3.93, 131**	**<0.001**

OR: odds-ratio, CI: confidence interval. Significant values are displayed in bold.
